# Are we chasing the wind? Translating global health commitments to actions, for health results

**DOI:** 10.4102/phcfm.v15i1.4148

**Published:** 2023-06-22

**Authors:** Humphrey C. Karamagi

**Affiliations:** 1 Office of the Assistant Regional Director, Regional Office for Africa, World Health Organization, Brazzaville, Congo

In the past decade, there have been several global health commitments. Among these are universal health coverage (UHC), health security (HSE), a revitalised primary health care (PHC) approach, determinants of health (DoH), and the sustainable development goals (SDGs).^1,2,3^ In practice, many frontline health workers receive these concepts with exasperation at the unending stream of guidance to which they need to align their activities. In addition, because of overlaps in interpretations, these aspirations have been mislabelled, misinterpreted, and generally abused by many actors in health, usually to cloak their pre-determined agendas. It is, therefore, common to see within countries, health systems championing specific interpretations of these concepts, leading to peculiarities such as a health programme ‘attaining UHC’, ‘health systems for a standalone program’, and others.^4,5,6,7^ It would appear, to an external observer, that the health sector is chasing the wind – recycling and creating new terms and initiatives to stay relevant.

However, when properly interpreted, these global commitments are individually important to ensure health services are responding to the current and future health and well-being needs of individuals and families. The nature of health has evolved, from a predominant focus on specific causes of ill health to include a more generalised individual need for well-being. An individual, even in a rural area, is not only concerned with avoiding dirty water and mosquitoes but also any issues that would hinder their social and/or economic productivity. The health sector is not only judged on whether it can reverse the effects of a mosquito bite (e.g. malaria treatment) but also on: (1) how this is achieved, (2) the fairness in ensuring everyone achieves this, and (3) the negative effects on the social and economic aspects of one’s life in achieving this. This should be done not only for mosquito bites but also for all issues that threaten a person’s feeling of health and well-being.

To achieve this, global commitments need to be tailored within each country, taking cognisance of the context, and the national authority’s need to be allowed to implement these. Two thrusts are needed for this to happen: (1) the global commitments need to be interpreted in an interlinked and complementary manner, so that (2) the countries can translate these within their context.

**FIGURE 1:**
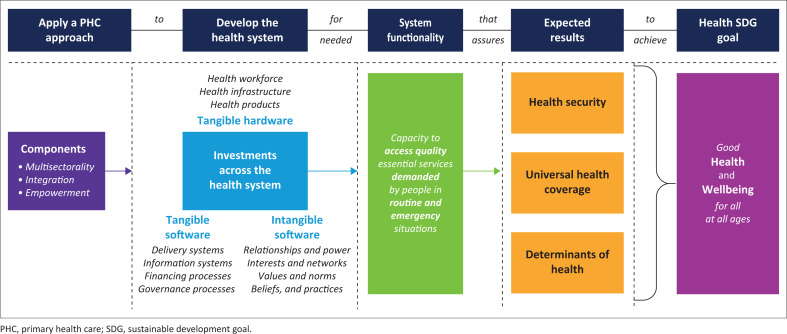
Rationalising the interlinkages amongst cross cutting global health committments.

To facilitate country interpretation, Figure 1 illustrates how these commitments are interlinked in a logical approach to facilitate the attainment of health and well-being that individuals and families are seeking.

The integrating question that countries should be asking, is ‘How should we apply the PHC approach to how we invest in the health system to attain the needed functionality of the health system that is necessary for maximising the utilisation of essential services that will achieve the goals of health in the SDGs?’ This brings together the global commitments in a logical, interlinked, and complementary manner that will ensure health expectations are met in countries. The level of flexibility in determining priorities increases as one moves from the right (SDG health goal) to the left (how the PHC approach is defined in a country).

The PHC approach is concerned with ‘how’ the three components of integration of services, empowerment of beneficiaries, and a multisectoral approach are applied when investing in the health system. The health system development focuses on investing in the elements that individuals and families interact with (tangible hardware), the elements the health bureaucracy prioritises for efficiency, equity, and effectiveness in using the hardware (the tangible software), and the subjective elements needed to nurture maximal productivity of assets (intangible software). The focus on functionality recognises that there are multiple correct ways to match the health system inputs, which makes the definition of standardised norms for investing in the health system impractical. Rather, countries should focus on ensuring the maximal capacity of the attributes shown to ensure functionality: access to essential services, quality of care, demand for essential services, and resilience to shocks.^8^ All countries are committed to achieving the results relating to UHC, HSE, and DoH,^9^ all of which lead to good health and well-being for all at all ages (SDG 3 goal).

As there is no single, normative path to responding to this integrating question, it needs to be asked in each country, with a national dialogue process to facilitate its translation. The national dialogue needs to explore questions of: (1) how should we plan and monitor components of integration, empowerment, and multisectorality while prioritising and making investments in the health system?; (2) How to determine which investments to make that will improve the existing functionality of the system?; (3) How will we measure and act on the functionality of the health system?; (4) What indicators will we use to monitor UHC, HSE, and health determinants? These questions need to be explored at the national level, mid-level, and frontline levels of health managers as the priorities are different for all. Additionally, for these commitments to be person-centred they need to be explored from the perspective of the individuals and families that benefit from the health system. The outcomes from such a dialogue form the basis for setting priorities in responding to global commitments at the country level.
